# Perioperative Antibiotics to Prevent Acute Endophthalmitis after Ophthalmic Surgery: A Systematic Review and Meta-Analysis

**DOI:** 10.1371/journal.pone.0166141

**Published:** 2016-11-08

**Authors:** Jinzhu Huang, Xiaofang Wang, Xiaohong Chen, Qiuyue Song, Wen Liu, Laichun Lu

**Affiliations:** 1 Department of Pharmacy, Daping Hospital, Third Military Medical University, Chongqing, China; 2 Department of Laboratory Medicine, Key Laboratory of Diagnostic Medicine (Ministry of Education), Chongqing Medical University, Chongqing, China; 3 Department of Pharmacy, Chongqing Health Center for Women and Children, Chongqing, China; 4 Department of Health Statistics, Third Military Medical University, Chongqing, China; 5 College of Pharmacy, Third Military Medical University, Chongqing, China; Save Sight Institute, AUSTRALIA

## Abstract

**Background:**

Post-operative endophthalmitis is a rare and dreaded complication in ophthalmic operations because it often induces irreparable vision loss. Although many ophthalmological studies aimed at reducing the rate of endophthalmitis have been performed around the world, controversy continues to surround some issues, including the choice of antimicrobials and their route of administration, duration and timing. The aim of this study is to investigate some of these unresolved issues.

**Methods:**

A systematic review and meta-analysis of randomized controlled trials and observational studies was performed. The PubMed, EMBASE, Cochrane Library and Clinical Trials databases were searched to identify studies published until Feb. 2016. The relative risk (RR) for each clinical outcome data is presented with 95% confidence intervals (CIs). Pooled estimates of effects were calculated using random-effect models.

**Results:**

Thirty-four studies from twenty-four reports involving 1264797 eyes were included in this analysis. Endophthalmitis occurred, on average, in one out of 6177 eyes when intracameral vancomycin/moxifloxacin were used and in one out of 1517 eyes when intracameral vancomycin/moxifloxacin were not used. The relative risk (95% CI) of endophthalmitis was reduced to 0.20 (0.10, 0.42) when intracameral antibiotics were used (p<0.0001). The subconjunctival injection of antibiotics was not superior to other administration routes included in this study (RR = 1.67, 95% CI (0.55, 5.05), p = 0.36). A statistically significant difference was found in the rate of endophthalmitis between the use and lack of use of topical antibiotics (RR = 0.65, 95% CI (0.43, 0.99), p = 0.04). However, no statistically significant difference was found in microbial isolation rates between these groups (RR = 0.77, 95% CI (0.34, 1.75), p = 0.53). When long-term and short-term use of topical antibiotics before surgery were compared, a statistically significant difference was found in microbial isolation rates (RR = 0.57, 95% CI (0.44, 0.74), p<0.0001).

**Conclusions:**

This meta-analysis concluded intracameral antibiotics are effective at preventing endophthalmitis in ocular surgery. A randomized controlled trial confirms the efficacy of cefuroxime but recent large cohort studies support the efficacy of vancomycin/moxifloxacin intracamerally. Intracameral antibitoics are superior to subconjunctival injections but that irrigation antibitoic data are not of enough quality to make a comparison. Different results were found in two clinical outcomes between the use or lack of use of topical antibiotic therapy, we did not find sufficient evidence to conclude that its use prevents endophthalmitis.

## Introduction

Post-operative endophthalmitis is a complication that can follow all ophthalmic procedures. Endophthalmitis is a calamitous event that can result in a patient suffering the loss of sight. Since 2000, the reported frequency of endophthalmitis is low worldwide, ranging from 0% to 0.63% [1]. Coagulase-negative *Staphylococcus* species are the organisms that are most frequently isolated from patients who develop postoperative endophthalmitis after cataract surgery. It is followed by coagulase-positive *Staphylococcus* species, *Streptococcus* species, *Enterococcus* species, and *Corynebacterium* species [2–7]. In pars plana vitrectomies, the reported bacteria include coagulase-negative *Staphylococcus* species, *Pseudomonas* species, *Propionibacterium* species, *Enterococcus* species, and *Bacillus* species [8]. Preoperative, intraoperative, and postoperative risk factors have been reported by some ophthalmologists. Diabetes mellitus, an immunocompromised state, chronic blepharitis, lacrimal passage infection, contaminated eyedrops, contact lens use, contralateral prosthesis and gender are preoperative factors, while the application of 2% xylocaine gel before povidone-iodine instillation, prolonged surgery, secondary surgery, posterior capsular rupture, vitreous loss and contaminated irrigating solution are intraoperative factors. Wound leakage, vitreous incarceration and behaviors (e.g., eye rubbing and personal hygiene) are associated with the development of post-operative endophthalmitis [9]. To reduce its incidence, many measures have been employed by ophthalmologists around the world. These include the use of topical antibiotics, intracameral antibiotics, subconjunctival antibiotic injections, lash trimming, saline irrigation, and antibiotic-containing irrigating solutions. However, the most frequent measure is the use of povidone–iodine before surgery to decrease contamination by ocular microbes and prevent postoperative endophthalmitis [10–12]. Not all of these techniques have been found to influence clinical outcomes. Intracameral cephalosporin (cefazolin, cefuroxime) has been shown to be effective in randomized controlled trials and meta-analyses [13–14]. An increasing number of ophthalmologists have supported the use of intracameral cefuroxime to prevent postoperative endophthalmitis because of the ESCRS study. However, as a result of the severe drug resistance of the causal bacteria, some new intracameral antibiotics, such as vancomycin and moxifloxacin, have been suggested to avoid endophthalmitis [15–21]. The subconjunctival injection and topical application of antibiotics have also been used by some ophthalmology centers, but the results of different reports have varied. This paper focuses on the use of intracameral vancomycin/moxifloxacin and the subconjunctival injection and topical application of antibiotics. The timing of topical drops was also analyzed. We sought to use a systemic review and meta-analysis to discuss those controversial issues.

## Materials and Methods

### Search Strategy

A comprehensive literature search was performed to identify studies published until Feb. 2016. The PubMed, EMBASE, Cochrane Library, and Clinical Trials databases were the main sources that were searched. Other routes (e.g., hand-search and library resource sharing) were also considered. Ophthalmologic surgical procedures, cataract extractions, vitrectomies, keratoplasties, intraocular lens implantations, glaucoma procedures, strabotomies, retinal detachment repair, laser in situ keratomileusis, laser-assisted subepithelial keratectomy, antimicrobial, antibacterial agents, antibiotic prophylaxis, anti-infective agent and eye surgery were the search terms that were used. The specific searching strategy is described in S1 Table.

### Inclusion and Exclusion Criteria

Studies were included if they met the following criteria: (i) random studies and observational studies, (ii) compared endophthalmitis rates or microbial isolation rates in two comparable populations, (iii) received/did not receive intracameral vancomycin/moxifloxacin therapy, or received/did not receive subconjunctival antibiotic injection, or received/did not receive topical antibiotics or compared administration with different timing, (iv) published from January 2000-February 2016 (reduced the influence of new operations), and (v) exceeded 1000 individuals if the study reported endophthalmitis rates and 50 if it reported microbial isolation rates. Studies were excluded if they: (i) were not written in English, (ii) were incomplete or included duplicated data, (iii) did not contain any predetermined clinical outcomes, (iv) could not be pooled with other included studies, (v) did not instill topical antibiotics (in terms of the group received topical antibiotics), and (vi) had no conformity at baseline with other studies in the timing (antibiotic drops and povidone–iodine were administered before surgery) and site of the analyzed specimen (conjunctival sac).

### Data extraction

The data were extracted independently by two authors (JZH and XHC). A standardized form was designed before the extraction to collect information including first author, publication date, mean age, male (%), type of surgery, study design, follow-up time, no. of eyes, therapeutic regimen, timing and clinical outcomes. If disagreements arose between these two authors, all final decisions were made by LCL and QYS after a discussion.

### Quality assessment

Randomized controlled trials (RCT) and observational studies were included in our analysis, and the quality assessment of those data is described in the S1 File. Observational studies were evaluated using the Newcastle–Ottawa scale (NOS) [22]. Nine items comprised the check list, and every item accounted for 1 point in each of three parts (selection, comparability and exposure). If a score was larger than 6, the study was determined to be of high quality. RCTs were assessed using the recommendations of the Cochrane Collaboration [23].

### Outcomes analyzed

In the current data set, the rate of endophthalmitis was the best and most direct clinical outcome that could be used to measure the effect of prophylactic antimicrobial agents. However, because postoperative endophthalmitis is rare, some studies also selected the microbial isolation rate as their outcome. In the analysis in this study, the rate of endophthalmitis was used as the primary outcome, and the microbial isolation rate was the secondary outcome.

### Statistical analysis

In this meta-analysis, we used risk ratios (RR) with 95% confidence intervals (CIs) to present dichotomous outcomes or enumeration data and random-effect models to calculate pooled estimates of effects. In light of fact that the rate of endophthalmitis was low, in case-control studies, the odds ratio (OR) was considered to be approximately equal to the RR. The I2 statistic was used to assess heterogeneity among studies. We considered the I2 values from 0% to 24%, 25% to 50% and greater than 50% to indicate low, moderate and high heterogeneity, respectively [23]. To decrease heterogeneity and increase reliability, a subgroup analysis was performed for every comparable group. Forest plots, the risk of bias in randomized controlled trials and the above-mentioned traits were analyzed using RevMan version 5.1. Furthermore, the analyses to determine sensitivity and publication bias analysis using Stata version 12.0.

## Results

### Results of the search and study characteristics

**Fig 1:**
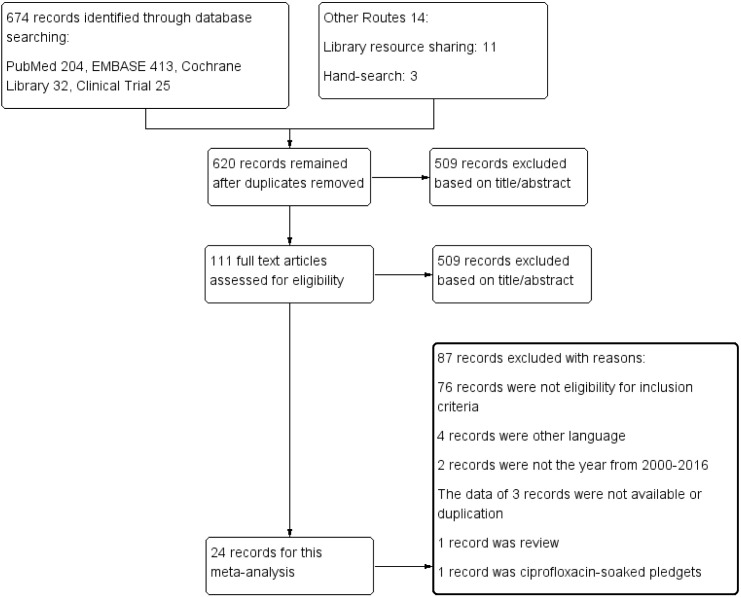
Flow diagram showing the selection process used to include studies in the meta-analysis.

Up to February 2016, a total of 688 reports were identified through database searches, and 34 studies included in 24 reports involving 1264797 eyes were included in the final analysis [2, 13, 15–21, 24–38]. The detailed screening process is described in Fig 1. Of the remaining reports, there were nine RCTs [13, 30–37] and fourteen observational studies [2, 15–21, 24–29, 38]. A total of 21 studies reported the rate of postoperative endophthalmitis [2, 13, 15–21, 24, 27–29, 38], and 13 studies reported the microbial isolation rate [25, 26, 30–37]. Thirty studies [2, 13, 15–21, 24, 27–30, 33–37, 38] included only eyes that received cataract surgery, but an additional four studies [25–26, 31–32] included other intraocular surgeries. The characteristics of the included studies are shown in Table 1.

**Table 1:** Main characteristics of the studies included in the meta-analysis.

**First Autdor, Date**	**Age**	**Male(%)**	**Type of Surgery**	**Study Design**	**Follow-up Time**	**No. of Eyes**		**Tderapeutic Regimen**		**Timing**		**Clinical Outcomes**	**Tde Quality of tde Study**
						**T**	**C**	**T**	**C**	**T**	**C**		
**Intracameral Antibiotic VS No Intracameral**													
Rush,2015	71.0	49.4	CAS	COS	3 months	9386	11333	IC vancomycin, PVI	PVI	PE	PE	RPE	High
Anijeet,2010	NA	NA	CAS	COS	6 weeks	12702	3904	IC vancomycin, PVI	PVI	PE	PE	RPE	Moderate
Rudnisky,2014	NA	NA	CAS	CCS	6 weeks	11818	59739	IC vancomycin	No intracameral antibiotic	PE	PE	RPE	Moderate
Rudnisky,2014	NA	NA	CAS	CCS	6 weeks	3738	59739	IC moxifloxacin	No intracameral antibiotic	PE	PE	RPE	Moderate
Shorstein,2013	74.0	NA	CAS	CCS	12 months	1890	3655	IC moxifloxacin, PVI	PVI	PE	PE	RPE	Moderate
Haripriya,2016	NA	NA	CAS	COS	6 weeks	38160	78554	IC moxifloxacin, PVI	PVI	PE	PE	RPE	High
Matsuura,2013	NA	NA	CAS	COS	1 month	18794	15958	IC moxifloxacin, PVI	PVI	PE	PE	RPE	Moderate
Friling,2013a	NA	38.3	CAS	COS	10 months	6897	2804	IC moxifloxacin, PVI	PVI	PE	PE	RPE	High
Galvis,2014	67.2	NA	CAS	COS	2 weeks	1618	1056	IC moxifloxacin, PVI	PVI	PE	PE	RPE	Moderate
**Subconjunctival Injection Antibiotic VS No Subconjunctival Injection**													
Jabbarvand,2016b	79.0	NA	CAS	RCS	6 weeks	69120	25290	SI antibiotic +PVI	IC+PVI	PE	PE	RPE	Moderate
Jabbarvand,2016c	79.0	NA	CAS	RCS	6 weeks	69120	76800	SI antibiotic +PVI	topical antibiotic +PVI	PE	PE	RPE	Moderate
Jabbarvand,2016d	79.0	NA	CAS	RCS	6 weeks	69120	260744	SI antibiotic +PVI	PVI	PE	PE	RPE	Moderate
Asencio,2015	71.5	NA	CAS	CCS	6 weeks	5068	9217	SI gentamicin +PVI	Irrigation BBS+ vancomycin+ gentamicin +PVI	PE	PE	RPE	High
Tan,2012	NA	NA	CAS	COS	1 month	29539	20638	SI gentamicin+ cefazolin +PVI	IC cefazolin +PVI	PE	PE	RPE	Moderate
Yu-Wai,2008	NA	NA	CAS	COS	3 weeks	19425	17318	SI cefuroxime +PVI	IC cefuroxime +PVI	PE	PE	RPE	Moderate
Colleaux,2000b	NA	NA	CAS	COS	NA	8856	5030	SI gentamicin ± cefazolin	PVI +Topical antibiotic	PE	PE	RPE	Moderate
**Topical Antibiotic VS No Topical Antibiotic**													
ESCRS,2007a	NA	NA	CAS	RCT	6 weeks	4000	3997	Topical levofloxacin +IC cefuroxime +PVI	Placebo +IC cefuroxime +PVI	PE	PE	RPE	High
ESCRS,2007b	NA	NA	CAS	RCT	6 weeks	3984	3990	Topical levofloxacin +PVI	Placebo +PVI	PE	PE	RPE	High
Coskun,2011a	NA	51.2	CAS	RCT	NA	54	53	Topical ciprofloxacin	PVI	PE	PE	MIR	Moderate
Coskun,2011b	NA	51.2	CAS	RCT	NA	57	53	Topical ofloxacin	PVI	PE	PE	MIR	Moderate
Eyal,2009	69.7	48.9	OCS	RCT	72 hours	237	227	Topical moxifloxacin +PVI	PVI	PE	PE	MIR	Moderate
**First Author, Date**	**Age**	**Male(%)**	**Type of Surgery**	**Study Design**	**Follow-up Time**	**No. of Eyes**		**Therapeutic Regimen**		**Timing**		**Clinical Outcomes**	**The Quality of the Study**
						**T**	**C**	**T**	**C**	**T**	**C**		
Kaspar,2008	67.8	34.1	OCS	RCT	10 days	67	65	Topical levofloxacin +PVI	PVI	PE	PE	MIR	High
Colleaux,2000a	NA	NA	CAS	COS	NA	12152	1734	Topical Tobramycin or Gentamicin or Ofloxacin or Polymyxin-trimethoprim	SI antibiotic +PVI	PE	PE	RPE	Moderate
Friling,2013b	NA	NA	CAS	COS	10 months	7307	396894	Topical chloramphenicol or fusidic acid	IC antibiotic +PVI	PE	PE	RPE	High
Jabbarvand,2016a	79.0	NA	CAS	RCS	6 weeks	76800	260744	Topical ciprofloxacin +PVI	PVI	PE	PE	RPE	Moderate
**Long time VS Short Time of Topical Antibiotic**													
Bing,2015	70.6	50.4	CAS	RCT	5 days	69	64	Topical neomycin/polymyxin-B	Topical neomycin/polymyxin-B	1d	1h	MIR	Moderate
Inoue,2008a	74.0	46.3	CAS	RCT	5 days	79	76	Topical levofloxacin	Topical levofloxacin	3d	1h	MIR	Moderate
Inoue,2008b	74.0	46.3	CAS	RCT	5 days	79	89	Topical levofloxacin	Topical levofloxacin	3d	1d	MIR	Moderate
Inoue,2008c	74.0	46.3	CAS	RCT	5 days	89	76	Topical levofloxacin	Topical levofloxacin	1d	1h	MIR	Moderate
Lingmin,2009	71.1	50.8	OCS	RCT	10 days	57	63	Topical moxifloxacin	Topical moxifloxacin	3d	1d	MIR	Moderate
Ta,2002	NA	NA	CAS	RCT	10 days	43	48	Topical ofloxacin	Topical ofloxacin	3d	1h	MIR	High
Ta,2007	NA	NA	CAS	RCT	6 days	50	50	Topical levofloxacin	Topical levofloxacin	3d	1d	MIR	High
Christopher,2008	69.3	68.3	OCS	COS	5 days	60	60	Topical moxifloxacin	Topical moxifloxacin	1d	1h	MIR	Moderate
Jason,2008	67.7	58.3	OCS	COS	5 days	60	60	Topical gatifloxacin	Topical gatifloxacin	1d	1h	MIR	Moderate

**Footnotes:**Age: Mean age or median age.

Abbreviations: NA = not available, T = treatment group, C = control group, CAS = cataract surgery, OCS = ocular surgery, COS = cohort study, CCS = case-control study, RCT = randomized controlled trial, RCS = retrospective cross-section study, IC = intracameral, SI = subconjunctival injection, PVI = povidone-iodine, PE = perioperation, 1d = 1 day before surgery, 3d = 3 days before surgery, 1h = within 1 hour before surgery, RPE = rate of postoperative endophthalmitis, MIR = microbial isolation rate.

### The Rate of Postoperative Endophthalmitis

#### Intracameral Antibiotic

**Fig 2:**
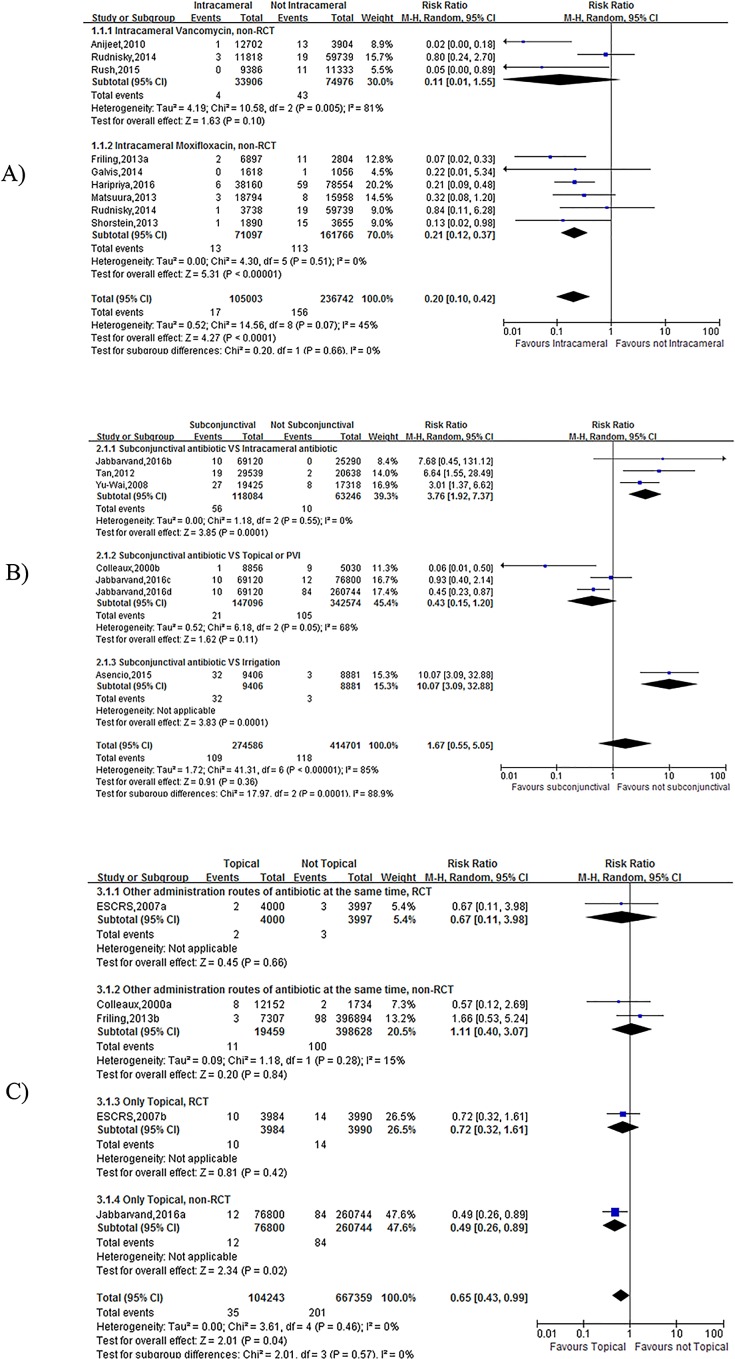
**Forest plot of the Rate of Postoperative Endophthalmitis (A: the effect of including and not including Intracameral Antibiotics; B: the effect of using VS not using subconjunctival antibiotic injections; C: the effect of using VS not using Topical Antibiotics).** The vertical line indicates no difference between the groups. RRs are represented by diamond shapes, and 95% CIs are depicted by horizontal lines. Squares indicate point estimates, and the size of each square indicates the weight of the given study in the meta-analysis. M-H, Mantel-Haenszel random-effects model.

Nine studies compared patients who received/did not receive intracameral vancomycin/moxifloxacin therapy [15–21, 38]. A significant difference was found in the meta-analysis results, which suggested that the rate of postoperative endophthalmitis was lower in the intracameral vancomycin/moxifloxacin group (OR = 0.20, 95% CI (0.10, 0.42), p<0.0001, I2 = 45%) (Fig 2A). In the subgroup analysis, the results in the moxifloxacin group was homologous (OR = 0.21, 95%CI (0.12, 0.37), p<0.00001, I2 = 0%) (Fig 2A). However, there was heterogeneity in the vancomycin group (OR = 0.11, 95%CI (0.01, 1.55), p = 0.10, I2 = 81%) (Fig 2A).

#### Subconjunctival antibiotic injections

Seven studies reported the use of subconjunctival antibiotic injections [2, 24, 27–29]. When patients who received subconjunctival antibiotic injections were compared to those who did not, no significant difference was found (OR = 1.67, 95% CI (0.55, 5.05), p = 0.36, I2 = 85%) (Fig 2B). Because I2 = 85%, three subgroup analyses were performed to increase reliability. The results suggested that a significant difference was found between the subconjunctival antibiotic injections group and the intracameral antibiotic and irrigation groups (intracameral antibiotic: OR = 3.76, 95% CI (1.92, 7.37), p = 0.0001, I2 = 0%; irrigation: OR = 10.07, 95% CI (3.09, 32.88), p = 0.0001) (Fig 2B). When the subconjunctival antibiotic injection group was compared to the topical or PVI (povidone-iodine) group, there was no significant difference (OR = 0.43, 95% CI (0.15, 1.20), p = 0.11, I2 = 68%) (Fig 2B).

#### Topical Antibiotic

The meta-analysis results revealed that there was a significant difference among the five studies (2, 13, 20, 24) that reported the rate of postoperative endophthalmitis (OR = 0.65, 95% CI (0.43, 0.99), p = 0.04, I2 = 0%) (Fig 2C). However, when subgroups were analyzed, no significant difference was found except for in a retrospective study (OR = 0.49, 95% CI (0.26, 0.89), p = 0.02) (Fig 2C).

### Microbial Isolation Rate

#### Topical Antibiotic

**Fig 3:**
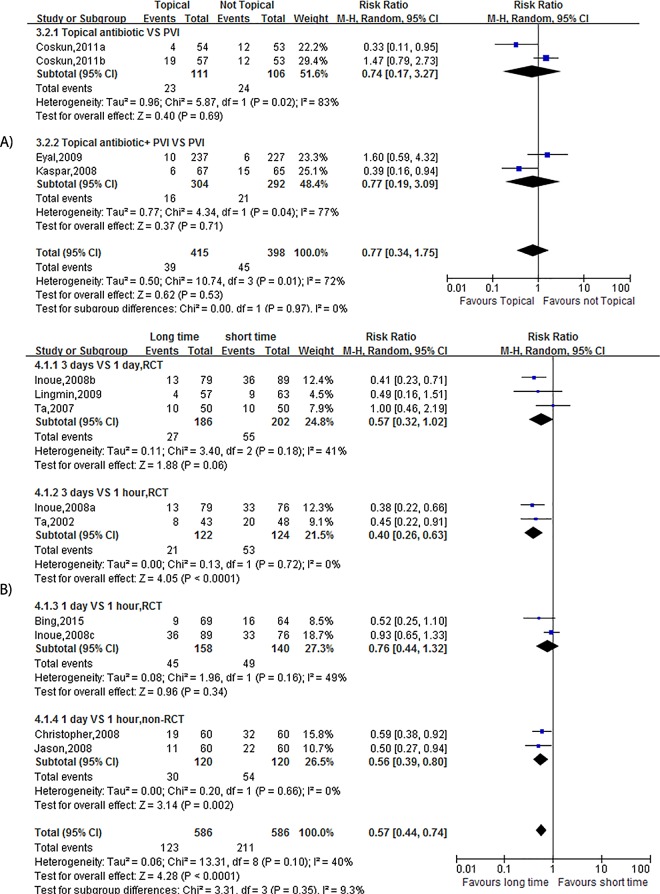
**Forest plot of the Microbial Isolation Rate (A: the effect of using VS not using Topical Antibiotics; B: the effect of Long-term VS Short-term use).** The vertical line indicates no difference between the groups. RRs are represented by diamond shapes, and 95% CIs are depicted by horizontal lines. Squares indicate point estimates, and the size of each square indicates the weight of the given study in the meta-analysis. M-H, Mantel-Haenszel random-effects model.

There were four studies [30–32] that provided a microbial isolation rate, and the results of these studies suggested that there was no significant difference (OR = 0.77, 95% CI (0.34, 1.75), p = 0.53, I2 = 72%) (Fig 3A). A subgroup analysis was performed, but the results were similar (only topical antibiotic: OR = 0.74, 95% CI (0.17, 3.27), p = 0.69, I2 = 83%; topical antibiotic + PVI: OR = 0.77, 95% CI (0.19, 3.09), p = 0.71, I2 = 77%) (Fig 3A).

#### Timing

When long-term and short-term use of a topical antibiotic before ocular surgery was analyzed, nine studies that had consistent baselines were included (25–26, 33–37). The meta-analysis revealed that short-term use was associated with an increased incidence of microbial isolation (OR = 0.57, 95% CI (0.44, 0.74), p<0.0001, I2 = 40%) (Fig 3B). Because there was moderate heterogeneity (I2 = 40%), a subgroup analysis was performed. A significance difference was found in the group that included two RCTs and that compared application between three days and within one hour before surgery (OR = 0.40, 95% CI (0.26, 0.63), p<0.0001, I2 = 0%) (Fig 3B). A significance difference was also found in the group that included two non-RCTs and that compared results between groups that were administered at one day and within one hour before surgery (OR = 0.56, 95% CI (0.39, 0.80), p = 0.002, I2 = 0%) (Fig 3B). There was no difference between the other two subgroups (3 days VS 1 day, RCT: OR = 0.57, 95% CI (0.32, 1.02), p = 0.06, I2 = 41%; 3 days VS 1 day, RCT: OR = 0.76, 95% CI (0.44, 1.32), p = 0.36, I2 = 49%) (Fig 3B).

## Publication Bias and Sensitivity Analysis

There was no significant publication bias according to the Begg’s and Egger’s funnel plot asymmetry tests that were performed in the meta-analysis (S2 File). The results of the sensitivity analysis were diverse, and the details are described in S3 File. In the group comparison between using and not using intracameral antibiotics, sensitivity was not influenced by the studies that were omitted obviously. The sensitivity analysis for receiving or not receiving subconjunctival antibiotic injections demonstrated that three studies (Colleaux,2000b, Jabbarvand,2016c and Jabbarvand,2016d) influenced the pooled effect size, and these studies were therefore deleted. New results that were similar to those of the subgroup analysis revealed that intracameral and irrigation antibiotics were superior to subconjunctival injections. In the case of topical antibiotics, we excluded the study (Jabbarvand, 2016a) because it influenced the sensitivity in the group that used RPE as a clinical outcome. When we then reanalyzed the data, we found that there was no statistical difference. Hence, that study should be deleted to increase the conclusion’s reliability. In the group in which MIR was used as the outcome, we re-conducted a subgroup analysis according to classifications of quinolone, but the result was similar to the previous result. The sensitivity analysis for the timing of application of topical antibiotics demonstrated that the study (Inoue, 2008c) exhibited slightly more influence on the pooled effect size than other studies, and this study was therefore deleted. The new result was almost the same as the initial result, but the heterogeneity was smaller. Because of the variety of available antibiotics, we also reanalyzed new subgroups according to the classifications of the drugs that were used. The result was similar to the previous result, but the heterogeneity was larger.

## Discussion

The low rate of postoperative endophthalmitis makes it difficult to conduct a large RCT to investigate the optimal method for preventing it [39]. Based on the currently available clinical evidence, preoperative preparation with 5% povidone-iodine solution has consensus approval among ophthalmologists. However, no consensus has been reached regarding the agent of choice, the administration route or the timing of antimicrobial prophylaxis, and no agent has been FDA-approved for this indication in ophthalmic procedures [1]. For these reasons, we performed this meta-analysis. However, because of the absence of relevant RCTs, we also included observational studies to resolve the problem mentioned above in this analysis. Since the ESCRS study, intracameral antibiotics have gradually gained acceptance around the world. Of those antibiotics, cefuroxime has been shown to be effective at reducing the rate of postoperative endophthalmitis. However, with the increase in resistant microorganisms, moxifloxacin and vancomycin have received an increasing amount of attention. The results of this meta-analysis show that intracameral moxifloxacin/vancomycin can prevent endophthalmitis. Unfortunately, no RCTs were included to address this point. Additionally, no study compared the use of cephalosporin with moxifloxacin or vancomycin. In addition to intracameral moxifloxacin/vancomycin, we also compared the results of administering subconjunctival antibiotic injections via other routes. According to our analysis, intracameral antibiotics were superior to subconjunctival injections, but subconjunctival injections were not superior to topical antibiotics or PVI. There is only one case-control study to compare irrigation antibiotics with subconjunctival injections, so the data is not enough quality to make a comparison. According to the results of this meta-analysis, we do not recommend subconjunctival antibiotic injections as a routine method for preventing endophthalmitis. Interestingly, when the timing of administration was not considered, topical antibiotics seemed to lose their value. The results were the same whether RPE or MIR was used as the clinical outcome. But there was a higher incidence of microbial isolation in short-term use than long-term use patients. The reason for this contradictory result might be that the efficiency of bacterial eradication varied with differences in the timing of topical antibiotics use. However, reducing the number of bacteria on the ocular surface is not the same as preventing endophthalmitis. Additionally, many topical antibiotics are used in combination, which makes it difficult to determine optimal timing [1]. The timing reported in the papers ranged from one hour to three days before the surgery [4, 11, 40–41]. Further investigations should be performed to optimize the best regimens. Given the results we obtained in the meta-analysis and the results from the ESCRS study, the use of topical antibiotics was not recommended.There are several limitations to our meta-analysis. First, few RCTs were included. Second, although we performed subgroup and sensitivity analyses, the I2 values remained large, especially in the topical antibiotic group, demonstrating that heterogeneity was high. The fact that we did not identify the reason for the heterogeneity may make the results unreliable. Third, some results were obtained from small sample studies. In addition, difficulties in diagnosing and defining endophthalmitis could also influence the primary data. For the studies that selected MIR as the clinical outcome, the method and duration of specimen cultivation might have made the results in the primary studies more diverse, which could have influenced our results.In conclusion, intracameral vancomycin/moxifloxacin therapy is effective for preventing postoperative endophthalmitis. Intracameral antibiotics are superior to subconjunctival injections, but that irrigation antibitoic data are not of enough quality to make a conclusion. Different results were found for topical antibiotic therapies between two clinical outcomes, and we did not find sufficient evidence to conclude that this technique prevents endophthalmitis. Long-term use of topical antibiotics before surgery appears to be more effective, but clearance the number of pathogens on the ocular surface is not the same as preventing endophthalmitis. So, topical antibiotics before surgery to prevent endophthalmitis were not recommended by this meta-analysis.

## Supporting Information

S1 PRISMA Checklist PRISMA Checklist for the meta-analysis. (DOC)

S1 File Quality assessment of included studies. (DOCX)

S2 File Publication bias. (DOCX)

S3 File Sensitivity analysis. (DOCX)

S1 Table Search strategy. (DOC)
