# Supplementary material for: Perioperative Antibiotics to Prevent Acute Endophthalmitis after Ophthalmic Surgery: A Systematic Review and Meta-Analysis
Source: PLoS One. 2016 Nov 8;11(11):e0166141. doi: 10.1371/journal.pone.0166141 (PMC5100907; doi:10.1371/journal.pone.0166141)
Supplement: S1 File — (DOCX) [file pone.0166141.s002.docx]

**S1 File. Quality assessment of included studies**

**Table1.** Quality assessment of included observational studies based on Newcastle-Ottawa scale

| Author, Year | Selection  (4 points) | Comparability  (2 points) | Exposure  (3 points) | Total  (9 points) |
| --- | --- | --- | --- | --- |
| Rush,2015 | 3 | 2 | 3 | 8 |
| Anijeet,2010 | 3 | 0 | 2 | 5 |
| Rudnisky,2014 | 3 | 0 | 3 | 6 |
| Shorstein,2013 | 3 | 0 | 3 | 6 |
| Haripriya,2016 | 3 | 1 | 3 | 7 |
| Matsuura,2013 | 3 | 0 | 3 | 6 |
| Friling,2013 | 3 | 1 | 3 | 7 |
| Galvis,2014 | 3 | 0 | 2 | 5 |
| Colleaux,2000 | 3 | 0 | 2 | 5 |
| Jabbarvand,2016 | 3 | 0 | 3 | 6 |
| Christopher,2008 | 4 | 1 | 3 | 8 |
| Jason,2008 | 4 | 1 | 3 | 8 |
| Yu-Wai,2008 | 3 | 0 | 3 | 6 |
| Tan,2012 | 3 | 0 | 3 | 6 |
| Asencio,2015 | 3 | 1 | 3 | 7 |

**Note:** We considered the value of total point from 1 to 3, 4 to 6, and ≥ 7 to indicate the quality of the study was low, moderate and high. Comparing with RCTs, the number of participants included in the observational studies was large (≥1000), we obeyed the above rules. On the contrary, if the number was less than 1000, we would make the final decision on Table1 to a lower level.

**Fig. 1** Risk of bias summary: review authors' judgements about each risk of bias item for randomized controlled trials
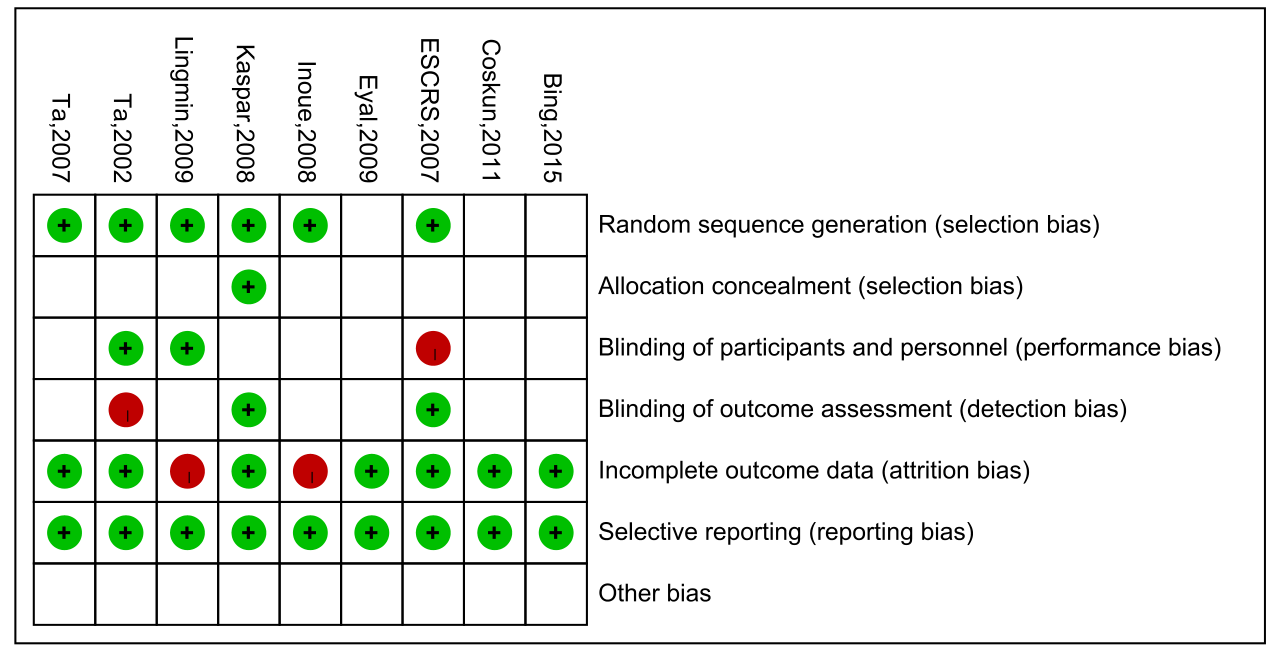


**Table2.** Quality appraisal of studies included in the meta-analysis.

| Study | Random  allocation | Concealment  schemes | Blinding | Withdrawals and Drop-out | | Jadad scale |
| --- | --- | --- | --- | --- | --- | --- |
| ESCRS,2007 | 2 | 1 | 1 | 1 | 5 | |
| Coskun,2011 | 1 | 1 | 1 | 1 | 4 | |
| Eyal,2009 | 1 | 1 | 1 | 1 | 4 | |
| Kaspar,2008 | 2 | 2 | 1 | 1 | 6 | |
| Bing,2015 | 1 | 1 | 1 | 1 | 4 | |
| Inoue,2008 | 2 | 1 | 1 | 0 | 4 | |
| Lingmin,2009 | 2 | 1 | 1 | 0 | 4 | |
| Ta,2002 | 2 | 1 | 1 | 1 | 5 | |
| Ta,2007 | 2 | 1 | 1 | 1 | 5 | |

**Note:**

We used Jadad scale to assess the included studies. Points were determined as follows,

1. Random allocation: computer-generated random numbers, 2 points; not described, 1 point; inappropriate method, 0 point.
2. Allocation concealment: central randomization, sealed envelopes or similar, 2 points; not described, 1 point; inappropriate or unused, 0 point.
3. Blindness: identical placebo tablets or similar, 2 point; inadequate or not described, 1 point; inappropriate or no double blinding, 0 point.
4. Withdrawals and drop-outs: numbers and reasons are described, 1 point; not described, 0 point.

The Jadad scale score ranges from 1 to 7; higher score indicates better RCT quality. If a study had a modified Jadad score >4 points, it was considered to be of high quality; if the score was 3-4 points, it was moderate quality; and if the score was <3 points, it was low quality.
