# Supplementary material for: Perioperative Antibiotics to Prevent Acute Endophthalmitis after Ophthalmic Surgery: A Systematic Review and Meta-Analysis
Source: PLoS One. 2016 Nov 8;11(11):e0166141. doi: 10.1371/journal.pone.0166141 (PMC5100907; doi:10.1371/journal.pone.0166141)
Supplement: S2 File — (DOCX) [file pone.0166141.s003.docx]

**S2 File.** Publication bias.

1. Intracameral Antibiotic VS Not Intracameral Antibiotic

1. Subconjunctival antibiotic injections VS Not subconjunctival

1. Topical Antibiotic VS Not Topical Antibiotic (RPE)

1. Topical Antibiotic VS Not Topical Antibiotic (MIR)

1. Timing of Topical Antibiotic

**Figure 1** Begg's funnel plot (each dot represents a separate study).

**Table1.** Egger's test of Fig 1A.

Std_Eff | Coef. Std. Err. t P>|t| [95% Conf. Interval]

slope | -.8246225 .8968099 -0.92 0.388 -2.945241 1.295996

bias | -.9803514 1.1798 -0.83 0.433 -3.770134 1.809431

p=0.433>0.05

**Table2.** Egger's test of Fig 1B.

Std_Eff | Coef. Std. Err. t P>|t| [95% Conf. Interval]

slope | -.4592269 1.327339 -0.35 0.743 -3.871261 2.952807

bias | 1.577633 2.581772 0.61 0.568 -5.059024 8.214289

p=0.568>0.05

**Table3.** Egger's test of Fig 1C.

Std_Eff | Coef. Std. Err. t P>|t| [95% Conf. Interval]

slope | -.8817271 .5454116 -1.62 0.204 -2.61747 .854016

bias | 1.025075 1.144629 0.90 0.436 -2.617646 4.667796

p=0.436>0.05

**Table4.** Egger's test of Fig 1D.

Std_Eff | Coef. Std. Err. t P>|t| [95% Conf. Interval]

slope | 4.714892 3.23873 1.46 0.283 -9.220237 18.65002

bias | -9.685013 6.342538 -1.53 0.266 -36.97475 17.60473

p=0.266>0.05

**Table5.** Egger's test of Fig 1E.

Std_Eff | Coef. Std. Err. t P>|t| [95% Conf. Interval]

slope | -.507148 .9553701 -0.53 0.612 -2.766239 1.751943

bias | -.7128556 2.293933 -0.31 0.765 -6.137145 4.711433

p=0.765>0.05

**Note:** The results of Begg’s and Egger’s funnel plot asymmetry tests in the meta-analysis indicated the absence of significant publication bias.
