# Supplementary material for: Perioperative Antibiotics to Prevent Acute Endophthalmitis after Ophthalmic Surgery: A Systematic Review and Meta-Analysis
Source: PLoS One. 2016 Nov 8;11(11):e0166141. doi: 10.1371/journal.pone.0166141 (PMC5100907; doi:10.1371/journal.pone.0166141)
Supplement: S3 File — (DOCX) [file pone.0166141.s004.docx]

**S3 File. Sensitivity analysis**

1. **Intracameral VS Not intracameral**

**Fig. 1A Sensitivity analysis of received intracameral antibiotic and not received**

**Table 1. Sensitivity analysis of received intracameral antibiotic and not received**

| Study omitted | Estimate | [95% Conf. Interval] | |
| --- | --- | --- | --- |
| Rush (2015) | 0.29835181 | 0.19219261 | 0.46314893 |
| Anijeet (2010) | 0.31847517 | 0.20392724 | 0.4973658 |
| Rudnisky (2014) | 0.24807805 | 0.15394071 | 0.39978197 |
| Rudnisky (2014) | 0.2701917 | 0.17270217 | 0.42271358 |
| Shorstein (2013) | 0.28993868 | 0.18548505 | 0.4532141 |
| Haripriya (2016) | 0.28014671 | 0.16488937 | 0.4759687 |
| Matsuura (2013) | 0.25796451 | 0.15943653 | 0.41738045 |
| Friling (2013) | 0.29173395 | 0.18353167 | 0.46372756 |
| Galvis (2014) | 0.27828775 | 0.17885701 | 0.43299432 |
| Combined | 0.28083259 | 0.18172983 | 0.43397907 |

**Note:** From the results of forest plot, there was moderate heterogeneity between each study (I^2^=45%, P=0.07). Sensitivity analysis demonstrated all studies did not influence the pooled effect size obviously.

1. **Subconjunctival antibiotic injections VS Not subconjunctival**

**Fig. 2A Sensitivity analysis of received subconjunctival antibiotic injections and not received**

**Table 2. Sensitivity analysis of received subconjunctival antibiotic injections and not received**

| Study omitted | Estimate | [95% Conf. Interval] | |
| --- | --- | --- | --- |
| Colleaux,2000b | 2.4803776 | 0.83791837 | 7.3423295 |
| Asencio,2015 | 1.2030548 | 0.40255833 | 3.5953569 |
| Jabbarvand,2016c | 1.8759485 | 0.47155478 | 7.4629353 |
| Jabbarvand,2016d | 2.2100645 | 0.68371331 | 7.1439083 |
| Yu-Wai,2008 | 1.4874271 | 0.3950371 | 5.6005863 |
| Tan,2012 | 1.3332939 | 0.40700986 | 4.36764 |
| Jabbarvand,2016b | 1.4520218 | 0.45415407 | 4.6424056 |
| Combined | 1.6698472 | 0.55252534 | 5.0466277 |

**Note:** From the results of forest plot, there was great heterogeneity between each study (I^2^=85%, P＜0.00001). Sensitivity analysis demonstrated three studies （Colleaux,2000b，Jabbarvand,2016c and Jabbarvand,2016d ） did influence the pooled effect size, so we deleted those studies. New results revealed intracameral or irrigation antibiotics were superior to subconjunctival injections (Fig. 2B).


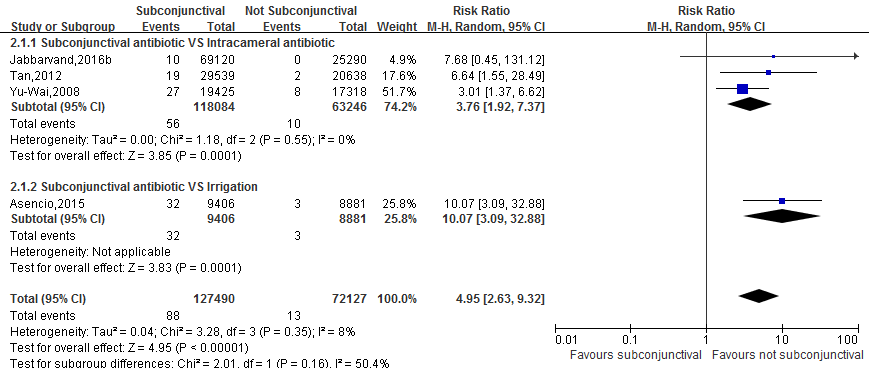


Fig. 2B The forest plot of remaining studies

1. **Topical Antibiotic VS Not Topical Antibiotic**
   1. **Topical Antibiotic VS Not Topical Antibiotic (RPE, the Rate of Postoperative Endophthalmitis)**

**Fig. 3-1A Sensitivity analysis of received topical antibiotic and not received, RPE**

**Table 3-1. Sensitivity analysis of received topical antibiotic and not received, RPE**

| Study omitted | Estimate | [95% Conf. Interval] | |
| --- | --- | --- | --- |
| Colleaux,2000a | 0.68194364 | 0.41674072 | 1.1159148 |
| Friling,2013b | 0.56426244 | 0.36039199 | 0.88346055 |
| ESCRS,2007a | 0.67218819 | 0.41265187 | 1.0949592 |
| ESCRS,2007b | 0.66381116 | 0.37451798 | 1.1765664 |
| Jabbarvand,2016a | 0.85104661 | 0.47775417 | 1.5160105 |
| Combined | 0.65088655 | 0.42866496 | 0.98830869 |

**Note:** From the results of forest plot, there was no heterogeneity between each study (I^2^=0%, P=0.46). Sensitivity analysis demonstrated study (Jabbarvand, 2016a) influenced the pooled effect size a little more than others, so we deleted it. New result revealed that no statistic difference was existent, and that was different from before (Fig. 3-1B).


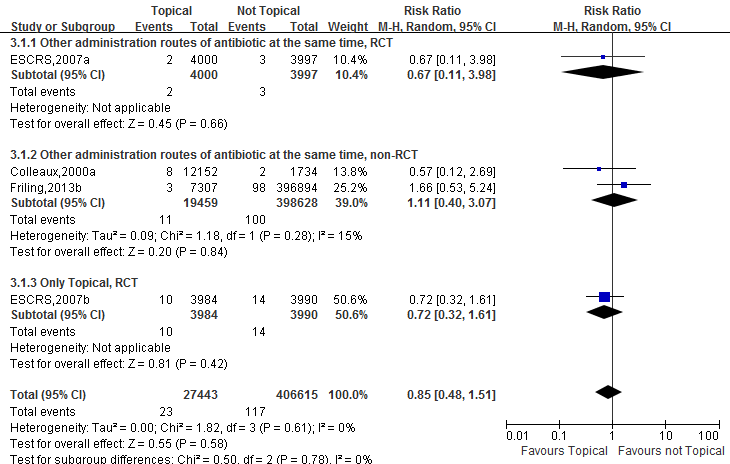


Fig. 3-1B The forest plot of remaining studies

- 1. **Topical Antibiotic VS Not Topical Antibiotic (MIR, Microbial Isolation Rate)**

**Fig. 3-2A Sensitivity analysis of received topical antibiotic and not received, MIR**

**Table 3-2. Sensitivity analysis of received topical antibiotic and not received, MIR**

| Study omitted | Estimate | [95% Conf. Interval] | |
| --- | --- | --- | --- |
| Coskun,2011a | 0.98647059 | 0.34949597 | 2.7843646 |
| Coskun,2011b | 0.53677754 | 0.17378046 | 1.6580123 |
| Eyal,2009 | 0.56170051 | 0.16521681 | 1.9096571 |
| Kaspar,2008 | 0.97092712 | 0.3302397 | 2.8545916 |
| Combined | 0.73724708 | 0.28037904 | 1.938566 |

**Note:** From the results of forest plot, there was great heterogeneity between each study (I^2^=72%, P=0.01). Sensitivity analysis demonstrated two studies （Coskun,2011a，Kaspar,2008） did influence the pooled effect size. Considering the variety of antibiotics, we reanalyzed by new subgroup. New results also revealed great heterogeneity, but the conclusions were almost the same with before (Fig. 3-2B).


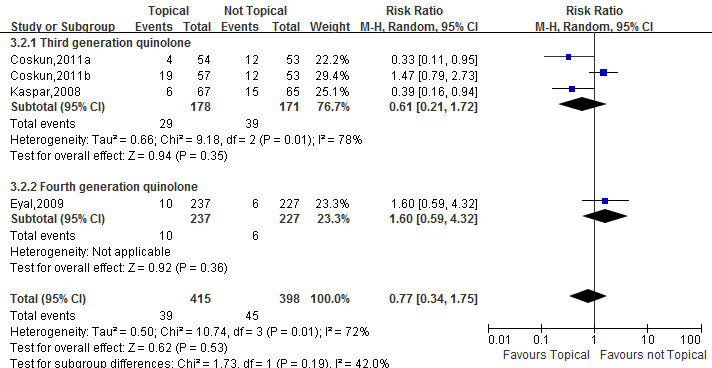


Fig. 3-2B The forest plot of new subgroup

1. **Timing of Topical Antibiotic**

**Fig. 4A Sensitivity analysis of received antibiotics long time and short time**

**Table 4. Sensitivity analysis of received antibiotics long time and short time**

| Study omitted | Estimate | [95% Conf. Interval] | |
| --- | --- | --- | --- |
| Bing,2015 | 0.44343427 | 0.30592713 | 0.64274768 |
| Inoue,2008a | 0.48414602 | 0.34680235 | 0.67588171 |
| Inoue,2008b | 0.47513857 | 0.3340861 | 0.67574395 |
| Inoue,2008c | 0.38282841 | 0.28282402 | 0.51819359 |
| Christopher,2008 | 0.44999735 | 0.30801549 | 0.65742671 |
| Lingmin,2009 | 0.44361656 | 0.30953427 | 0.63577984 |
| Ta,2002 | 0.45937184 | 0.32062349 | 0.65816289 |
| Jason,2008 | 0.45169837 | 0.31132967 | 0.65535487 |
| Ta,2007 | 0.41479958 | 0.30200894 | 0.56971391 |
| Combined | 0.44485657 | 0.31996742 | 0.61849223 |

**Note:** From the results of forest plot, there was small heterogeneity between each study (I^2^=40%, P=0.10). Sensitivity analysis demonstrated study (Inoue, 2008c) influenced the pooled effect size a little more than others, so we deleted it. New result was almost the same with before (Fig. 4B), but heterogeneity was smaller than before (I^2^=0%, P=0.64). Considering the variety of antibiotics, we also reanalyzed new subgroup, but the heterogeneity was bigger than the former (I^2^=40%, P=0.10) (Fig. 4C).


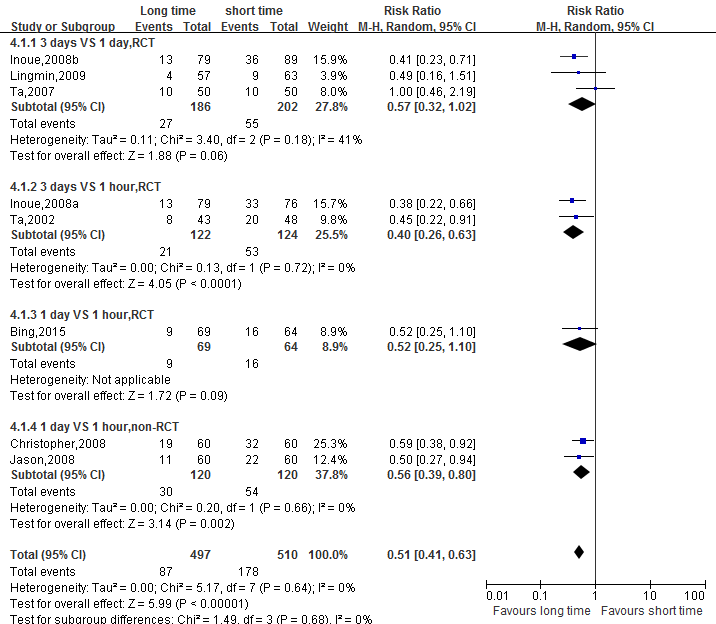


Fig. 4B The forest plot of remaining studies


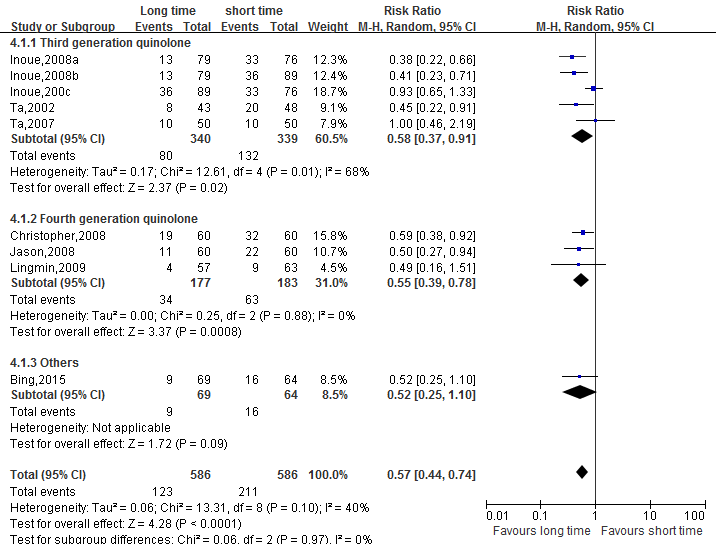


Fig. 4C The forest plot of new subgroup
