# Supplementary material for: Perioperative Antibiotics to Prevent Acute Endophthalmitis after Ophthalmic Surgery: A Systematic Review and Meta-Analysis
Source: PLoS One. 2016 Nov 8;11(11):e0166141. doi: 10.1371/journal.pone.0166141 (PMC5100907; doi:10.1371/journal.pone.0166141)
Supplement: S1 Table — (DOC) [file pone.0166141.s005.doc]

**S1 Table.** Search strategy.

| **PubMed:** 204 records (up to February 2016)  (((((((((((((ophthalmologic surgical procedures[MeSH Terms]) OR cataract extractions[MeSH Terms]) OR vitrectomies[MeSH Terms]) OR keratoplasties[MeSH Terms]) OR intraocular lens implantations[MeSH Terms]) OR glaucoma procedures[MeSH Terms]) OR strabotomies[MeSH Terms]) OR retinal detachment repair[MeSH Terms]) OR laser in situ keratomileusis[MeSH Terms]) OR laser-assisted subepithelial keratectomy[MeSH Terms])) AND (((antimicrobial[MeSH Terms]) OR antibacterial agents[MeSH Terms]) OR antibiotic*[MeSH Terms]))) AND antibiotic prophylaxis [MeSH Terms] |
| --- |
| **EMBASE:** 413 records (up to February 2016)  #4 #1 AND #2 AND #3  #3 'antibiotic prophylaxis'/exp  #2 'antiinfective agent'/exp  #1 'eye surgery'/exp |
| **Cochrane Library:** 32 records (up to February 2016)  #1 MeSH descriptor: [Ophthalmologic Surgical Procedures] explode all trees  #2 MeSH descriptor: [Anti-Infective Agents] explode all trees  #3 MeSH descriptor: [Antibiotic Prophylaxis] explode all trees  #4 #1 and #2 and #3 |
